# Review of Modelling Techniques for *In Vivo* Muscle Force Estimation in the Lower Extremities during Strength Training

**DOI:** 10.1155/2015/483921

**Published:** 2015-08-31

**Authors:** Florian Schellenberg, Katja Oberhofer, William R. Taylor, Silvio Lorenzetti

**Affiliations:** Institute for Biomechanics, ETH Zurich, HCI E 351, 8093 Zurich, Switzerland

## Abstract

*Background*. Knowledge of the musculoskeletal loading conditions during strength training is essential for performance monitoring, injury prevention, rehabilitation, and training design. However, measuring muscle forces during exercise performance as a primary determinant of training efficacy and safety has remained challenging. *Methods*. In this paper we review existing computational techniques to determine muscle forces in the lower limbs during strength exercises *in vivo* and discuss their potential for uptake into sports training and rehabilitation. *Results*. Muscle forces during exercise performance have almost exclusively been analysed using so-called forward dynamics simulations, inverse dynamics techniques, or alternative methods. Musculoskeletal models based on forward dynamics analyses have led to considerable new insights into muscular coordination, strength, and power during dynamic ballistic movement activities, resulting in, for example, improved techniques for optimal performance of the squat jump, while quasi-static inverse dynamics optimisation and EMG-driven modelling have helped to provide an understanding of low-speed exercises. *Conclusion*. The present review introduces the different computational techniques and outlines their advantages and disadvantages for the informed usage by nonexperts. With sufficient validation and widespread application, muscle force calculations during strength exercises *in vivo* are expected to provide biomechanically based evidence for clinicians and therapists to evaluate and improve training guidelines.

## 1. Introduction

The quantification of muscle forces during muscle strengthening exercises* in vivo* has tremendous potential for assisting with training design, performance monitoring, and injury prevention [1]. Due to the fact that 45.6% of all injuries during strength training in Switzerland occur due to overloading [2] and current exercise guidelines as well as training recommendations are based on the subjective experience of individual experts or coaches, knowledge about the effects of external loading on internal muscle forces during training or rehabilitation could help to improve exercise safety. In addition, the analysis of the internal loading conditions provides an evidence-based approach for defining specific targets and loading goals for effective training outcome while also reducing injury risk, since fewer loads can be used to achieve the same training effect. Furthermore, muscles are able to specifically adapt to their loading output to the surrounding functional requirements and must therefore respond appropriately to allow the rehabilitation of unbalanced musculature in an effective and safe way. However, measuring muscle forces* in vivo* remains challenging due to the complexity of movement control, the nonlinear material properties of muscle tissue, the redundant number of muscle actuators, and the invasive nature of direct measurement techniques [3]. Existing guidelines on strength training (type of exercise, repetitions, number of sets, etc.) are often based on experience or simple measurements from dynamometry or surface electromyography (EMG) [4–7], but the actual stress levels in the muscle based on muscle force and cross-sectional area measures, which provide direct evidence for the efficacy and safety of specific muscle-strengthening exercises, have been difficult to obtain [8, 9].

It is not currently possible to measure muscle forces experimentally during exercise performance* in vivo*, and data from alternative measurement techniques have not been sufficient for deducing internal forces and moments for complex dynamic systems, such as the lower limbs, in a straight forward manner [10]. Common measurement techniques in human motion analysis and sports science include surface EMG, optical motion capture, and force measures, for example, dynamometer or force platforms [8]. Dynamometry has frequently been used to determine strength and power during open chain leg extension or flexion exercises [6]. However, strength and power are scalar variables that provide only limited insights into actual muscle forces that occur internally, especially considering the complexity of inter- and intramuscular activity and coordination associated with free weights or multijoint dynamic training [6]. On the other hand, surface EMG provides a good insight into muscle activation levels during functional exercise performance compared to strength and power measures gained from dynamometry. However, while EMG offers more specific information on muscle function compared to dynamometry, it still provides insufficient data to deduce muscle force magnitudes [7], especially when analysing dynamic movements [11].

**Figure 1:**
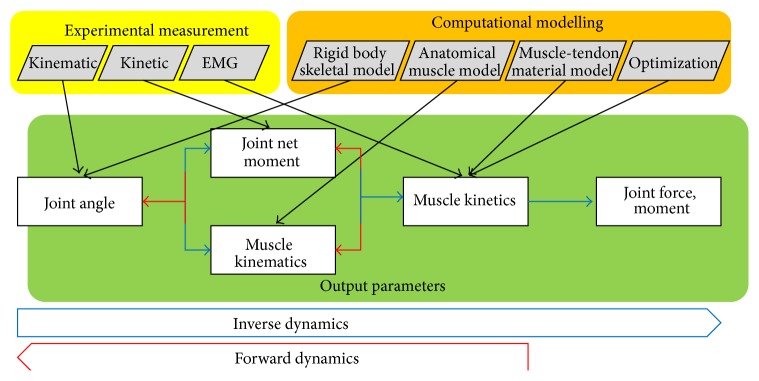
Muscle and joint forces are quantified* in vivo* by combining experimental measurements (yellow) with computational biomechanics (orange). Different measurement parameters (black arrows) or computational optimizations (black arrows) are required to achieve different output parameters (green) in inverse dynamics or forward dynamics processes. For forward dynamics simulations (red arrows), usually applied to dynamic ballistic movement exercises such as the squat jump, joint dynamics such as joint angles, joint net moment, or muscle kinematics are derived by finding an optimal set of muscle kinetics using computational modelling. For inverse dynamics analysis (blue arrows), usually applied to low-speed exercises such as the squat, joint moments, muscle forces, and finally joint contact forces are derived from joint angles and net joint moments.

Computational models of the musculoskeletal system are therefore needed to provide a link between externally measured data and internal forces and moments (Figure 1). Musculoskeletal modelling techniques have been developed and extensively used in clinical and biomechanical gait analysis, in particular for studying lower limb dynamics. In order to predict muscle forces during movement, a computational model has to capture the anatomy of the musculoskeletal system, as well as the physiological force generating properties of muscle tissue, and then relate the target movement to the internal muscle forces through Newton's laws of motion [13]. Additional parameters, such as individual ratios of fast-twitch versus slow-twitch fibres within each muscle or muscle versus fat volumes within the segments, could be taken into account in an optimization process and enhance the accuracy of a model but also its complexity (Figure 1).

Depending on model complexity, available experimental data, and study goal, the dynamic system of equations (originating from Newton's second law of motion) can be solved in different ways, including forward dynamics [12, 14–20], inverse dynamics [7, 13, 21–24], and EMG-driven analyses [4, 5, 25, 26]. Using forward dynamics, a set of muscle activation patterns are usually chosen as input into a physiological muscle model to derive muscle forces. Muscle forces are then applied to a rigid body skeletal model to estimate joint moments or joint angles (Figure 1). In contrast, inverse dynamics uses data from experimental measurements including skin marker positions and ground reaction forces as input into a rigid body skeletal model to calculate joint net moments. In addition, using optimization processes and musculoskeletal modelling, joint forces and moments can be computed from the results of the inverse dynamics analysis. An EMG-driven analysis uses normed muscle activation levels from EMG measurements in addition to skin marker positions and ground reaction forces to improve the estimation of muscle force magnitudes by means of musculoskeletal modelling. Unfortunately, the unknown muscle forces that cause a particular movement generally exceed the known parameters from experimental measurements, resulting in redundant systems of equations that require the use of various optimization techniques [4, 12, 20, 24, 26] (Figure 1).

Improved knowledge of the specific muscle forces that act during strength training could help coaches and athletes improve training protocols, as well as physiotherapists and patients to undertake rehabilitation exercises in an efficient and safe manner. Furthermore knowledge of the muscle forces can be used as boundary conditions within continuum organ models in order to estimate the biological change of the tissues such as muscle, bone, and tendon due to the mechanical stimuli of strength training. However, current approaches for deriving muscle forces are generally complex and require substantial expertise in computational modelling. In this review of the literature, we introduce nonexperts to current musculoskeletal modelling techniques for determining muscle forces (generally of the lower limbs) during strength training* in vivo* and discuss their potential as well as limitations for application to sports practice in order to assist with training recommendations and guidelines. In this manner, this review aims to result in an improved understanding of the existing computational techniques and thus provide a basis for future developments and more widespread and informed application of available biomechanical tools. Eventually, the results of in-depth biomechanical analyses are expected to help define objective, evidence-based guidelines for coaches and therapists to execute strength training exercises in an effective and safe manner.

## 2. Methods

A systematic electronic literature search of the US National Library of Medicine was conducted in August 2013. Four combined concepts including different search parameters were used to systematically search the literature (Table 1). A total of 77 papers were found complying with all four concepts and were further assessed for eligibility to be included in the review based on the following exclusion criteria: (1) no measurements were taken during a functional strength exercise of the lower extremities, or (2) results were limited to EMG data, maximum voluntary isometric contraction, or net joint moments without adopting a computational model to determine muscle forces, or (3)* ex vivo* study. Out of these 77 papers, a total of 12 articles were eligible for the full review. Articles were mainly excluded because results were limited to measured data from EMG or dynamometry, without adopting musculoskeletal modelling techniques to determine muscle forces or other internal forces. Additional studies were excluded because muscle forces were analysed during activities other than strength training or were* ex vivo*, performed on cadaveric specimens. Strength training was defined as a physical exercise that induces muscular contraction to enhance strength, anaerobic endurance, or size of a skeletal muscle. References from the included papers were further searched for relevant work including the same criteria. An additional 9 papers were found within the references and included in the review process. Ultimately, the full text articles of 21 studies were included in the review.

## 3. Results

Musculoskeletal models with different levels of anatomical detail and computational complexity have been developed to determine muscle forces during strength exercises of the lower extremities* in vivo* (Table 2). The 21 included studies were divided into three categories, with 9 studies performing forward dynamics simulation, 2 studies adopting quasi-static inverse dynamics optimisation, and 10 studies outlining mixed inverse/forward dynamics (1 study) or mixed inverse dynamics/alternative methods including EMG-driven modelling (9 studies) (Table 2). Forward dynamics simulations have predominantly been used to study dynamic ballistic movement exercises such as the squat jump, while quasi-static inverse dynamics optimisation techniques or alternative methods have been adopted to analyse low-speed exercises such as the lunge or leg press under the assumption of “negligible acceleration in each segment.” Although Pearsall and Costigan [30] showed that accurate net moments may be achieved using quasi-static approaches in low-speed exercises, some studies have used a true inverse dynamics approach with inclusion of the accelerations of the segments and their moments of inertia [31–34]. All methodologies have in common that they draw on the physics principles of multibody dynamics (Newton's laws of motion) and rely on accurate representations of musculoskeletal anatomy and physiology to accurately predict the muscle forces that cause the target motion (Figure 1).

### 3.1. Anatomical and Physiological Model Parameters

Musculoskeletal models with different numbers of muscles and joints, and different material property characteristics for active muscle and passive soft tissues, have been introduced depending on the research goal, available data, and expertise in computational modelling. Musculoskeletal models with a reduced number of muscles and/or a lower number of degrees of freedom at the joints have been adopted to simplify analyses. In particular, the knee joint has generally been represented as a planar hinge joint, neglecting translational and rotational degrees of freedom other than flexion-extension [7, 12, 13, 17, 20, 21, 24, 26, 28, 29]. In these models, individual muscles have often been grouped to reduce the unknown degrees of freedom of a musculoskeletal model, such as the hamstrings or quadriceps [17, 20, 21, 24, 25], or only key muscle players have been considered [7, 27]. Exceptions are the anatomical models adopted by different authors [13, 28, 29], which considered up to 43 muscle-tendon units per leg. These authors additionally subdivided large or complex muscles such as the gluteal muscles into multiple muscle units to more accurately represent their muscle paths and functions than would single muscle units [13]. The anatomical and physiological properties of these three models were based on experimental measurements of cadaveric specimens from the literature in an attempt to represent the modelled subject-specific properties [29]. Two of the models were generically implemented into commercially available or open-source software packages (LifeModeler, OpenSim) [13, 28].

**Figure 2:**
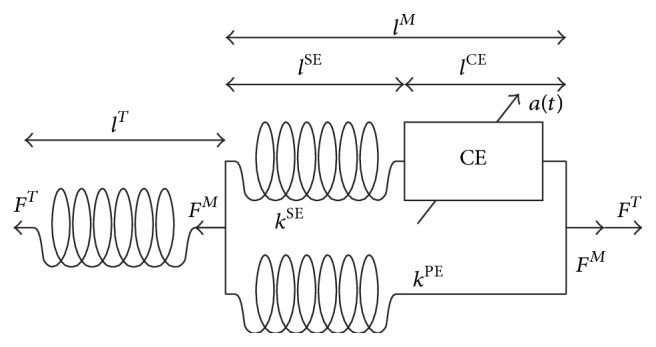
The Hill-type muscle-tendon model, showing muscle and tendon forces (*F* ^*M*^, *F* ^*T*^), as well as the series-elastic (SE), parallel-elastic (PE), and contractile (CE) elements of the muscle length (*l*) and stiffness (*k*) of the whole muscle-tendon actuator (*M*, *T*). *a*(*t*) represents the activation of the CE (adapted from Pandy and coworkers [12]).

The active and passive material properties of muscle-tendon structures have commonly been described by a Hill-type [35] active element, which comprises a so-called contractile element (CE) (force generation by actin and myosin cross-bridges) to capture the force-length-velocity dependency of muscle tissue. This active element is additionally coupled with two passive elements (represented by nonlinear spring elements); a series-elastic (SE) element to account for the tendon elasticity and a parallel-elastic element (PE) to account for the passive stiffness of the muscle connective tissue (Figure 2) [12, 14–20, 29]. The force-velocity dependency of a particular muscle can thus be derived directly from Hill's equation (*v* + *b*)(*F* + *a*) = *b*(*F*_0_ + *a*) [35], where larger forces can be produced by the CE during slow velocity contractions and vice versa. During eccentric movement, muscles are able to produce even higher forces, since passive structures additionally support the CE for force production. The force that can be produced by a muscle is further dependent on the actual length of a muscle, since the force generated actin myosin cross-bridges at the sarcomere level depend on their overlapping status. Furthermore passive structures (PE and SE) produce force when the muscle is stretched, even if the CE is not activated. The Hill-type muscle model has widely been accepted by the biomechanics community and has been implemented into musculoskeletal modelling software packages such as OpenSim [10, 36].

Material parameters (predetermined, constant values) for the Hill-type muscle model are generally derived from experimental measurements on cadaveric specimens reported in the literature, including maximum isometric force, muscle fibre pennation angle, tendon slack length, tendon and muscle passive stiffness, and physiological cross-sectional area (PCSA). Simplifications to the Hill-type model have been made by neglecting the force-velocity and/or force-length relationship as well as the passive material properties, especially to analyse quasi-static exercises using inverse dynamics techniques [13, 21–24, 28]. Adjustment of material parameters to individual subjects is generally achieved through simple scaling based on segmental lengths, calculated joint centers, EMG signals (normalized to the maximal voluntary isometric contraction (MVIC)), or subjects body weight [4, 5, 12, 18–24]. Alternatively, static or functional optimisation approaches can be used [26]. Other techniques to determine individual muscle parameters include ultrasound measurements, which allow the evaluation of muscle volume and thus PCSA [3], but no studies have been found that combine ultrasound measurements with musculoskeletal modelling to analyse muscle forces during strength training.

### 3.2. Forward Dynamics Simulation

In general, the problem to simulate a musculoskeletal model using a forward dynamics approach is to find a physiologically feasible set of controls regarding the muscle activity, for example, by means of the minimized integral cost function. This usually includes a large set of boundary conditions and constraints to be defined. A set is related to different application area such as movement, loading conditions, physiology, and the time dependency of an optimization algorithm. Furthermore, forward dynamics simulations are usually associated to a control problem. Here, open loop solutions are often highly unstable and difficult to integrate while close loop solutions of the highly nonlinear musculoskeletal system remain still an unsolved problem. Significant attempts have been made to simulate the musculoskeletal system in motion based on forward dynamics, using muscle activation levels as input and the time-history of segmental positions and orientations as output, in particular to study dynamic ballistic movement exercises such as the squat jump. Here, different studies have introduced forward dynamics models of the musculoskeletal system to better understand, for example, how intermuscular control [12, 14, 18], bilateral-asymmetry [15, 29], or muscular fatigue [16] affect the maximum jump height. The dynamic equations of motion of the skeletal system are thereafter described by a set of differential equations, driven by muscle-tendon actuators which are controlled by neural signalling. Muscle-tendon actuators are commonly represented using the Hill-type muscle-tendon model, connected to a numerical model to capture the time lapse between the incoming neural signal and the onset of muscle activation. Forward dynamics simulations depend on optimisation algorithms to find feasible sets of muscle activation patterns leading to the desired movement dynamics, with maximum jump height as a common performance criterion. For other movements such as squats or lunges, new criteria would need to be defined. Solutions to the optimisation problem are more likely found for unambiguous movement patterns with simplified musculoskeletal models that are with a limited number of muscle actuators and constrained conditions such as reduced degrees of freedom of the joints [12].

**Figure 3:**
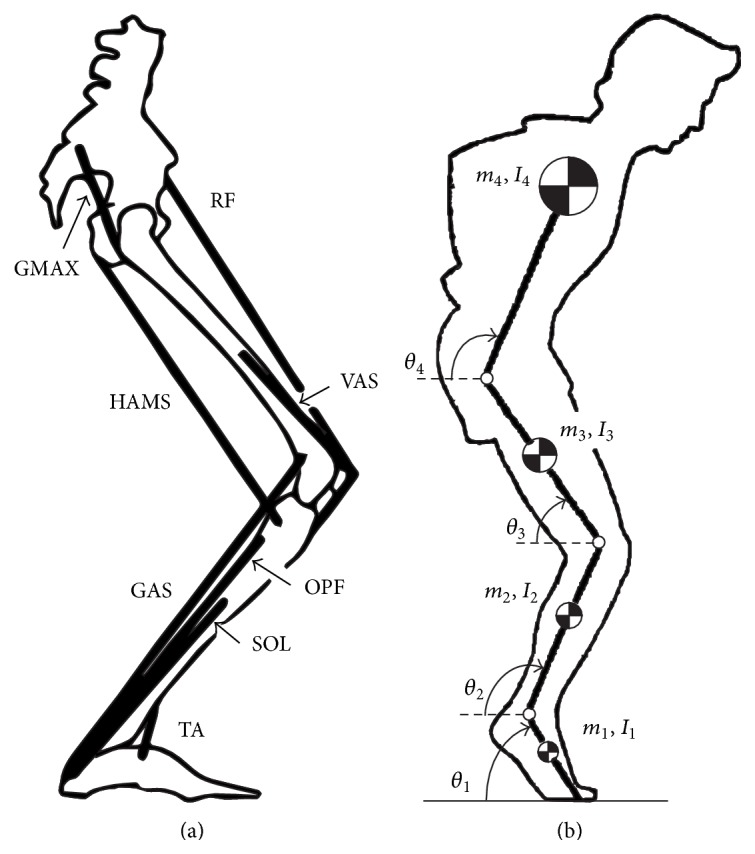
(a) Schematic representation of the musculoskeletal model for the vertical jump and (b) the four-segment multibody model with lumped masses and mass moments of inertia for the foot, shank, thigh, and head/arms/trunk (Pandy and coworkers [12]).

One of the first forward dynamics models for the planar squat jump was introduced by Pandy and coworkers [12], comprising all lower limb bones and eight major muscles (Figure 3). The constraints that defined the optimal control problem were the dynamic equations of motion, the terminal calculation point at takeoff, and the muscle activation levels being set to 0 or 1, with maximum jump height as the performance criterion. A more restricted form of dynamic optimisation was introduced by van Soest and coworkers [20], whereby muscles were only allowed to switch from the initial activation values once and then had to maintain maximum activation until takeoff. The problem was thus reduced to finding an optimal combination of the muscular switching times to result in maximum jump height. The resulting muscle activation patterns corresponded well with experimentally measured data from EMG, and the formulation has often been applied to biomechanical analyses of the squat jump [14–18].

In order to gain confidence in forward dynamics simulations, modelling results have often been compared with experimental data from optical motion capture, force platforms, and EMG [15–17, 19, 20], confirming the ability of forward dynamics models to accurately reproduce the major features of maximum-height squat jumps. Based on the present literature search, and in agreement with previous reviews on muscle force calculations in orthopaedics and clinical gait analysis [36], forward dynamic methods have not yet been applied to muscle strengthening exercises other than the squat jump. While the performance criterion for the squat jump is generally maximal jump height, the selection of performance criteria for other activities is considered more challenging [36]. Furthermore, forward dynamic models require multiple integration steps to reach optimal joint kinematics, resulting in a computational complexity that limits their implementation in user-friendly software packages and thus widespread use by nonexperts. However, using forward dynamics offers the possibility for coaches or therapists to simulate an optimal training or rehabilitation program for a specific athlete or patient without the need for elaborate experimental measurements such as EMG, optical motion capture, or ground reaction forces.

### 3.3. Quasi-Static Inverse Dynamics Optimisation

In contrast to forward dynamics simulation, the inverse dynamics formulation is comparably quick and computationally inexpensive. Inverse dynamics analysis refers to the calculation of segmental forces and moments based on data from optical motion capture and force sensors such as force platforms and has become a routine tool in clinical gait analysis [36] and strength training exercises [37–40]. It is important to note that joint contact forces and muscle forces cannot be calculated directly from inverse dynamics. Instead, the derivation of muscle forces necessitates distributing the net intersegmental forces from inverse dynamics across synergistic and antagonistic muscles, which leads to a problem of indeterminate nature that needs to be solved using numerical optimisation techniques. Joint contact forces can additionally be calculated as the sum of the net intersegmental forces and the synergistic and antagonistic muscle forces that cross the joint (Figure 1).

Quasi-static inverse dynamics optimisation techniques have generally been applied to low-speed movements, such as the leg press or lunge [7, 13, 21, 22, 24, 27]. For low-speed exercises, the quasi-static equilibrium condition holds true under the assumption that the angular acceleration of each segment is negligible. In an early study, Reilly and Martens [27] introduced a single-muscle model to quantify quadriceps muscle forces during deep knee bends based on inverse dynamics. The model worked under the assumption that only the quadriceps as a single muscle group is active during the exercise. Thus, the net knee joint moments from inverse dynamics were equal to the resulting muscle moments, and muscle forces could be determined geometrically by deriving the moment arms with respect to the knee joint centre. A similar modelling approach was adopted by Henriksen and coworkers [7], analysing the eccentric and concentric forces in the Achilles tendon during ankle plantar- and dorsiflexion. Here, the advantage is that single-muscle models do not depend on computationally expensive optimisation techniques; however, the potential contribution of synergistic and antagonistic muscles to joint stability and movement control is neglected and the physiological differences in force generating capabilities between muscles cannot be accounted for.

More complex musculoskeletal models based on quasi-static inverse dynamics have been developed, accounting for the contribution of synergistic and antagonistic muscle groups to analyse open and closed chain knee extension [21–23] and hip flexion-extension [13]. Here, optimisation algorithms based on the least-squares method have generally been adopted to find weighting factors for each muscle force contribution to minimize the differences between the intersegmental torques from inverse dynamics and the resultant muscle torques from the biomechanical model. In early attempts, muscle forces were assumed to be proportional to physiological cross-sectional area (PCSA), maximum voluntary contraction force, and measured EMG activation levels, without taking the force-length [24], muscle fibre recruitment [7], and force-velocity [21, 23] relationships into account. To improve results, Zheng and coworkers [24] extended previous models by examining the role of muscle force-length properties and demonstrated that force-length dependent optimisation during squat and leg press exercises had a significant effect on muscle force magnitudes, which proved to be an important factor in determining tension in the cruciate ligaments.

A slightly different approach based on quasi-static inverse dynamics optimisation was adopted by Lewis and coworkers [13] to analyse the effect of position and alteration in synergist muscle forces on hip forces during hip strengthening exercises. Muscle-tendon paths and maximum isometric forces of 43 muscle units were adopted from a generic musculoskeletal model in the commercially available software SIMM (MusculoGraphics, Inc., Santa Rosa, CA, USA). Muscle material properties other than muscle-tendon paths and maximum isometric force were neglected, including force-length relationships and the passive response to stress. An optimisation algorithm was adopted that aimed to minimise muscle stress with the goal of maximizing muscle endurance. Such an approach has been widely accepted for biomechanical analyses of the lower limbs during gait [36]. However, quasi-static optimisation techniques based on minimizing muscle stresses have been shown to underestimate antagonistic muscle activity as well as muscle force contributions of low magnitudes [41]. Furthermore, subjects who are fatigued or in pain are unlikely to activate muscles according to a minimal effort principle but rather with the aim of avoiding mechanical stress on fatigued or painful tissue.

### 3.4. Alternative Methods

A group of alternative methods have been introduced to calculate muscle forces based on a mixed inverse-forward dynamics approach [28] or by using EMG data to drive a musculoskeletal model towards given joint kinematics (EMG-driven modelling) [4, 25, 26]. In particular, Nolte and coworkers [28] outlined a combined inverse-forward dynamics simulation to quantify intervertebral loading during the abdominal crunch exercise based on a full-body musculoskeletal model using the LifeModeler software. The modelling output depended on the initial estimation of muscle-tendon lengths from inverse dynamics to provide reference values for the derivation of muscle activation levels and thus muscle forces. However, the model did not account for physiologically realistic material properties of muscle tissue. Instead, muscle forces were derived using a closed loop algorithm containing proportional-integral-differential controllers to reach the target length-time curve. Despite questions arising in terms of model validity, the study remains unique in that it included a Computer Aided Design (CAD) model of the training machine. By using musculoskeletal models of different sizes together with the CAD model, the authors were able to analyse the effectiveness and safety of the exercise machine to accommodate very small or large individuals based on the predicted muscle forces and intervertebral joint loading.

Other alternative methods to determine muscle forces during strength training include so-called EMG-driven musculoskeletal models, introduced by Lloyd and Besier [26] and Escamilla and coworkers [4, 5]. The basic concept behind EMG-driven models consists of collecting EMG data, which are filtered, rectified, and input into a calibrated musculoskeletal model to predict muscle forces. However, extensive calibration trials, including optical motion capture and ground reaction force measurements, are required to define subject-specific model parameters before accurate predictions of muscle forces for individual subjects across a number of different tasks are possible. Calibration trials allow the adjustment of model parameters by minimizing the differences between joint kinematics and/or joint torques from inverse dynamics analysis and corresponding results from the EMG-driven model. In contrast to quasi-static inverse dynamics optimisation techniques, EMG-driven models account for the dynamic force generating properties of muscles and, upon successful calibration, have been adopted to predict muscle forces during dynamic exercises such as sidestepping or dynamic lunge activities [4, 5]. Challenges remain in the placement of the electrodes and processing of the EMG signals as well as in the calibration of reference or generic musculoskeletal models to subject- and task-specific conditions [3]. Despite these challenges, high density EMG measurements have been shown to reduce errors in muscle force predictions, but these analyses often result in an instrumentation complexity that may not be achievable during sports practice [3].

## 4. Discussion

The relevance of understanding the internal muscle forces during strength exercises becomes clear when examining the wide range of studies and research questions reported in the literature. Musculoskeletal modelling techniques have been applied to strength training to analyse the effect of altered muscle physiology on exercise performance (physiological adaptations) [14, 17, 20], the impact of exercise execution on muscle and joint forces (best choice of exercise to avoid injury) [4, 5, 21–23], and the internal loading state of differently sized people using the same exercise machines (safety and efficacy of equipment) [28]. Muscular weaknesses, bilateral asymmetries, or changes in exercise performance have been shown to result in altered and potentially harmful internal tissue loading that cannot be investigated based on external observation or simple measurements.

Accurate assessment of the risks involved in strength exercises, and subsequent design of effective exercise schemes, is dependent on the accurate estimation of muscle forces and joint loading during the target exercise. Different numerical techniques have been introduced to determine muscle forces in the lower extremities during strength training* in vivo*, including (1) forward dynamics analysis to study dynamic ballistic movement exercises such as the squat jump, (2) quasi-static inverse dynamics optimisation to study low-speed exercises, such as the lunge or leg press, and (3) alternative methods such as EMG-driven modelling. All methodologies are challenged by the limitations of externally measurable data and the complexity of the musculoskeletal system, that is, the indeterminate nature of the simulation problem. Forward dynamics analyses depend on optimisation algorithms to find the most suitable set of muscle activation levels that lead to the desired movement patterns, while muscle force calculations from inverse dynamics analyses require optimisation algorithms to distribute the net joint moments across synergistic and antagonistic muscles in a physiological manner.

The findings by studies presented in this review have provided insights into the biomechanical principles underlying strength training that would not otherwise be possible. Using musculoskeletal modelling techniques, health care related factors could be detected. For example, co-contraction forces of the hamstrings and quadriceps during squats and leg presses have been shown to significantly affect tension in the cruciate ligaments [21–23], which in turn is a crucial factor for establishing safe and effective rehabilitation programs. Furthermore, the results from musculoskeletal modelling have provided sports performance related factors, such as the muscle activation delays between stimulation onset times of proximal muscles versus plantar flexors during the squat jump and their influence on jump height deficits [18]. Lastly, the knowledge gained from computational studies has helped to support and establish training and injury prevention recommendations. For example, the reduced quadriceps muscle forces during long step lunges compared to short step lunges have supported the belief of clinicians and trainers that anterior knee translation beyond the toes during the forward lunge may be harmful to the patellofemoral joint [4].

Essentially, the forward dynamics simulation is a method of systematic trial and error and could represent the process by which an athlete optimizes control of muscle recruitment and physiological strength for best performance of explosive movements such as the squat jump [12]. Predictive analyses based on forward dynamics provide a powerful tool to elucidate the impact of alterations in neurological activation, muscular physiology, or joint alignment on performance output. As such, predictive analyses have considerable potential to improve strength training guidelines, without the need for extensive experimental measurements on individual subjects. Compared to forward dynamics simulation, quasi-static inverse dynamics optimisation techniques are computationally efficient and do not depend on EMG measurements. In particular, quasi-static optimisation techniques based on maximising muscle endurance (minimizing stress) have been widely accepted to estimate muscle forces in the lower extremities during walking and stair climbing [36, 42]. Application of the same techniques to strength training may be valid for quasi-static exercises when the training goal is maximising strength endurance. However, static optimisation techniques are generally not sufficient for predicting antagonistic muscle activity that does not occur with the goal of minimizing stress but rather to stabilize joints and maintain joint integrity [43]. EMG-driven models provide an alternative to static optimisation techniques, especially to determine muscle forces following injury or muscular fatigue where muscle recruitment patterns might be altered. However, EMG-driven models are challenged by extensive validation trials to formulate valid muscle model parameters, and difficulties remain in the placement of EMG electrodes, signal normalization, and filter choice.

Unfortunately, it remains challenging to confirm the validity of musculoskeletal models for accurately reproducing muscle forces due to the invasive nature of internal measurement techniques. Other than, for example, tendon-force measurements during surgery [36] or telemetric implants [42, 44], a gold standard for model validation remains lacking. To address this issue, an international consortium of biomechanics researchers has received funding from the National Institutes of Health to organise a series of five “Grand Challenge Competitions to Predict* In Vivo* Knee Loads” [45]. The goal is for competitors to predict the* in vivo* medial and lateral knee contact forces for specific movement trials collected from subjects implanted with a force-measuring tibial prosthesis. Muscle forces are the primary determinants of joint contact forces, and thus instrumented implant data provide a direct validation of joint contact forces and an indirect validation of muscle forces. The validation of musculoskeletal models by means of instrumented joint implants has proven invaluable in orthopaedic research, for example, as a basis for standardizing preclinical testing [42]; however, instrumented implants have exclusively been used in elderly subjects to analyse joint loading during daily activities such as walking or stair-climbing and have therefore not been applied to training and sports problems that involve dynamic ballistic movement loading conditions and/or impact.

It is important to note that the required degree of model complexity is dependent on the particular research question. Simplifications regarding motion, anatomy, and physiology are often required in order to reduce the computational costs. However, simplified musculoskeletal models with generic material properties may lead to invalid results for certain cases. For example, the knee abduction angle during jump landing tasks was shown to be a predictor of anterior cruciate ligament injury risk in female athletes [46], whereby all rotational degrees of freedom should be involved in the knee joint during rehabilitation from anterior cruciate ligament injury; or the relationship between the measured EMG signal and the actual muscle force of the triceps surae was shown to be different for the eccentric versus the concentric contraction phase of one-legged full weight bearing ankle plantar and dorsiflexion exercises [7]. These studies suggest that all rotational degrees of freedom in the joints, or contraction-specific EMG-to-force relationships, may be required to improve modelling results in specific cases. Ideally, the influence of model complexity on the simulation results is assessed prior to making any conclusions.

Limitations of musculoskeletal simulations remain in the accurate capturing of subject-specific anatomy (e.g., segment lengths, degrees of freedom, and muscle paths) and physiology (e.g., Hill-type muscle, force-length, and force-velocity relationships). As a result, anatomical and physiological parameters such as the PCSA of muscles have mainly been adopted from cadaveric measurements on a few elderly human subjects (5 cadaver specimens, mean age 79.2 years in Herzog and Read [47], or 2 male cadaver specimens, mean age 82 years in Spoor and coworkers [48]) and scaled to subject-specific dimensions based on a few measurements. Lloyd and Besier [26] introduced more extensive techniques to calibrate an EMG-driven model to subject-specific conditions; however, the physiological basis of the calibration process was questioned and seemed rather tedious for application to sports practice. In the broader field of human motion analysis, increased efforts have been directed towards developing efficient computational techniques to create subject-specific anatomical models based on magnetic resonance images [49, 50]. The future application of such techniques to strength training could certainly provide a basis for analysing the influence of individual differences in muscle physiology and anatomy on exercise performance. Furthermore, subject-specific customisation of muscle model properties may be based on supplementary parameters from ultrasound or dynamometry. Such parameters could include the type of muscle fibre, maximal isometric or dynamic muscle force, or physiological performance parameters such as maximal power.

Substantial efforts have been directed towards translating musculoskeletal modelling techniques into user-friendly tools to facilitate their application in clinical and sports practice. In particular, the open source software OpenSim, developed and maintained on https://simtk.org/ by the NIH National Center for Physics-Based Simulation of Biological Structures (Simbios, Stanford University, CA, USA), has significantly contributed to the uptake of computational biomechanics by the nonexpert [10]. OpenSim provides a user-friendly interface for coupling forward dynamics, quasi-static inverse dynamics, and EMG-driven modelling with subject-specific experimental data to calculate joint and muscle dynamics during human movement. The development of easy-to-use and freely available software such as OpenSim marks a significant step towards the more wide-spread application of advanced computational techniques for improving the efficacy and safety of strength training. Interestingly, only a few studies have reported actual muscle force values, even though many have outlined computational techniques that entailed the calculation of muscle forces (Table 2). It appears that muscle forces are often used only as intermediate parameters to analyse, for example, maximal jump height through forward dynamics simulation or quantify joint forces and moments through inverse dynamics analyses. As a result, it seems that the potential of muscle force calculations to provide evidence for the efficacy and safety of strength training programmes has not yet been fully grasped, possibly due to the difficulties in validating the presented solutions, thus explaining the limited confidence for uptake.

Knowledge of the acting muscle forces during strength training based on musculoskeletal modelling offers a means to establish effective and safe training guidelines to achieve specific training aims such as improved intermuscular coordination, monitoring muscular changes, eliminating or preventing unbalanced muscular adaptation, pointing out injury predictors, and improving efficiency and safety of exercise equipment. In the future, it might be possible to model subject-specific loading conditions during exercising and further use the predicted muscle forces as boundary conditions for finite element continuum models in order to estimate the biological tissue adaptation due to training. The application of existing musculoskeletal modelling techniques to strength training of the lower limbs is particularly attractive because of standardised conditions and simple movement patterns that are often associated with strength exercises. A key factor in limiting the applicability and uptake of the musculoskeletal modelling techniques to clinical and sports practice seems to be the lack of experimental solutions for validating internal forces, stresses, and strains* in vivo*. Cross-institutional initiatives, like the grand challenge to determine* in vivo* knee loads, should be extended to include more subjects in different age groups and a range of different activities including strength exercises. Upon successful model validation, the quantification of muscle forces during strength training based on inverse and forward dynamic analyses is expected to help improve current training guidelines to the benefit of coaches, clinicians, athletes, and patients alike.

## Figures and Tables

**Table 1:** Four concepts of search parameters were used to systematically search the literature and were combined using an “and” condition (horizontal). Each concept was created using “or” conditions (vertical) in order to ensure the inclusion of all papers using similar definition for the same case.

MeSH		Concept number 1		Concept number 2		Concept number 3		Concept number 4
			*and *		*and *		*and *	
Subject [MeSH]	*or *	Resistance training		Musculoskeletal system		Lower extremity		Computer simulation^*^
				Muscle, skeletal		Leg		
						Ankle		
						Knee		
						Hip		
						Foot		

Text words [title/abstract]	*or *	Strength training		EMG		Lower limb		Muscle force
		Weight-lifting		Electromyogra^*^		Lower body		Muscle stress
		Weight-bearing				Lower extremities		Muscle control
		Strengthening						Musculoskeletal modeling
								Musculoskeletal modelling
								Optimization
								Optimisation
								Simulation
								Forward dynamic simulation
								Computer models

^*^They were used as wildcards to replace part of a string.

**Table 2:** Summary of studies reporting on computational techniques to determine muscle forces during strength training of the lower extremities *in vivo*. Dynamic squat jump was mainly analysed using forward dynamic (FD) simulation, while low-speed ankle, hip, and knee exercises were analysed using quasi-static inverse dynamics (ID) optimisation, electromyography-driven (EMG) modelling, or mixed inverse dynamics/forward dynamics analysis. Different approaches were adopted to distribute the net joint moments from ID across muscles, ranging from simple 1-muscle models to advanced optimization schemes taking muscle force-length-velocity (*F*-*l*-*v*) into account. Data from EMG, optical motion capture (OMC), and ground reaction forces (GRF) were used as input or reference to assess the accuracy of modelling results.

	Exercise	Modelling approach	Subjects	Experi. measure	Reported results	Reference
Low-speed	Foot plantar/dorsi flexion	ID (1-muscle model)	8 M, 8 F (22 y)	EMG, OMC, GRF	Muscle force	Henriksen et al. (2009) [7]
	Deep knee bends	ID (1-muscle model)	3 M (26 y)	OMC, GRF	Muscle and joint forces	Reilly and Martens (1972) [27]
	Squat, leg press, knee extension	ID (*F*-EMG)	10 M (30 y) experienced	EMG, OMC, GRF	Tibiofem joint kinetics, cruciate ligament force	Wilk et al. (1996) [23], Escamilla et al. (1998, 2001) [21, 22]
	Squat, leg press	ID (optimized *F*-*l*-EMG)	10 M (30 y) experienced	EMG, OMC, GRF	Tibiofem joint kinetics, cruciate ligament force	Zheng et al. (1998) [24]
	Squat, leg press, knee extension	ID (optimized *F*-*l*-*v*-EMG)	9 M (29 y), 9 F (25 y), low body fat	EMG, OMC, GRF	Patellofemoral force and stress	Escamilla et al. (2008) [4, 5]
	Squat	EMG-driven/ID/FEM	8 M (29 y), 8 F (29 y)	EMG, OMC, GRF, MRI, open MRI	Knee cartilage stress	Besier et al. (2008) [25] based on Lloyd and Besier (2003) [26]
	Hip extension/flexion	ID (min. stress)	Generic simul.	—	Hip joint forces	Lewis et al. (2009) [13]
	Abdominal crunch	Mixed ID/FD equipment	Generic simul. (three anthropometric cases)	—	Intervertebral joint loading	Nolte et al. (2013) [28]

Dynamic ballistic	Squat jump	FD (activation *a*_initial or *a* = 1)	6 M (25 y) well-trained volleyball players	EMG, OMC, GRF	Gastro bioarticularity	van Soest et al. (1993) [20]
			6 M (25 y) well-trained volleyball players	EMG, OMC, GRF	Muscle strengthening	Bobbert and Van Soest (1994) [17]
			6 M (25 y) well-trained volleyball players	EMG, OMC, GRF	Triceps surae series elastic compliance	Bobbert (2001) [14]
			Generic simul.	—	Stimulation onset times	Bobbert and van Zandwijk (1999) [18]
			6 M (26 y)	EMG, OMC, GRF	Fatigue of plantarflexors	Bobbert et al. (2011) [16]
			8 M (20 y) well-trained volleyball and gymnastics	EMG, OMC, GRF	Bilateral deficit	Bobbert et al. (2006) [15]
		FD (activation *a* = 0, 1)	Generic simul.	—	Optimal controls	Pandy et al. (1990) [12]
			5 M (22 y)	EMG, OMC, GRF	Contribution of muscles to accelerate trunk	Pandy and Zajac (1991) [19]
		FD (n/a)	Generic simul.	—	Bilateral asymmetry	Yoshioka et al. (2011) [29]
